# Systemic osteosclerosis associated with primary bone marrow B‐cell lymphoma

**DOI:** 10.1002/jha2.652

**Published:** 2023-02-05

**Authors:** Shuji Ozaki, Tsutomu Iima, Etsuko Sekimoto, Hironobu Shibata, Toshio Shigekiyo, Yukio Higuchi, Takanori Hirose

**Affiliations:** ^1^ Department of Hematology Tokushima Prefecture Central Hospital Tokushima Japan; ^2^ Department of Cardiovascular Medicine Tokushima Prefecture Central Hospital Tokushima Japan; ^3^ Department of Orthopedic Surgery Tokushima Prefecture Central Hospital Tokushima Japan; ^4^ Department of Diagnostic Pathology Tokushima Prefecture Central Hospital Tokushima Japan

**Keywords:** osteoprotegerin, osteosclerosis, primary bone marrow B‐cell lymphoma

## Abstract

Systemic osteosclerosis is a rare complication of hematological malignancies. Primary myelofibrosis and acute megakaryocytic leukemia are known as underlying diseases; however, lymphoid tumors have rarely been reported. Here we describe a case of a 50‐year‐old man with severe systemic osteosclerosis associated with primary bone marrow B‐cell lymphoma. Analysis of bone metabolic markers revealed a high turnover of bone metabolism and an increase in serum osteoprotegerin levels. These results suggest the involvement of osteoprotegerin in the pathogenesis of osteosclerosis associated with hematological malignancies.

## 1 INTRODUCTION

Osteosclerosis is a rare disease of skeletal abnormalities with diffuse radiographic appearance of high bone density due to increased bone formation and/or suppressed bone resorption, and is caused by congenital or secondary disorders such as metabolic and neoplastic diseases [1]. In hematological malignancies, primary myelofibrosis [2] and acute myeloid leukemia [3], especially, acute megakaryocytic leukemia [4, 5] are known as underlying diseases. In lymphoid tumors, osteosclerosis occurs occasionally in Hodgkin's lymphoma [6], but rarely in non‐Hodgkin's lymphoma except for a few cases of lymphocytic lymphoma [7] and hairy cell leukemia [8, 9, 10].

Primary bone marrow lymphoma (PBML) is a unique type of lymphoid malignancy originating in the bone marrow [11]. In PBML, lymphoma cells are diffusely spread in the bone marrow and can be distinguished from primary bone lymphoma that forms localized bone tumors [12]. We experienced a rare case of severe systemic osteosclerosis associated with PBML.

## 2 CASE REPORT

A 50‐year‐old man was referred to our hospital because of pancytopenia. He had a 4‐year history of bilateral hip joint pain. Physical examination revealed moderate anemia but no lymphadenopathy. Laboratory examination showed a hemoglobin concentration of 7.3 g/dl (reference range, 13.5–17.6 g/dl), a white blood cell count of 2.5 × 10^9^/L (3.9–9.8 × 10^9^/L) with neutrophils of 0.9 × 10^9^/L, lymphocytes of 1.4 × 10^9^/L, and monocytes of 0.11 × 10^9^/L, and a platelet count of 74 × 10^9^/L (135–367 × 10^9^/L). Serum lactate dehydrogenase was normal at 173 IU/L (110–220 IU/L), but alkaline phosphatase (ALP) was elevated at 518 IU/L (110‐350 IU/L). Serum calcium, phosphate, and parathyroid hormone were within normal range. The serum level of soluble interleukin‐2 receptor (sIL‐2R) was markedly elevated at 5380 U/L (124–466 U/L).

**FIGURE 1:**
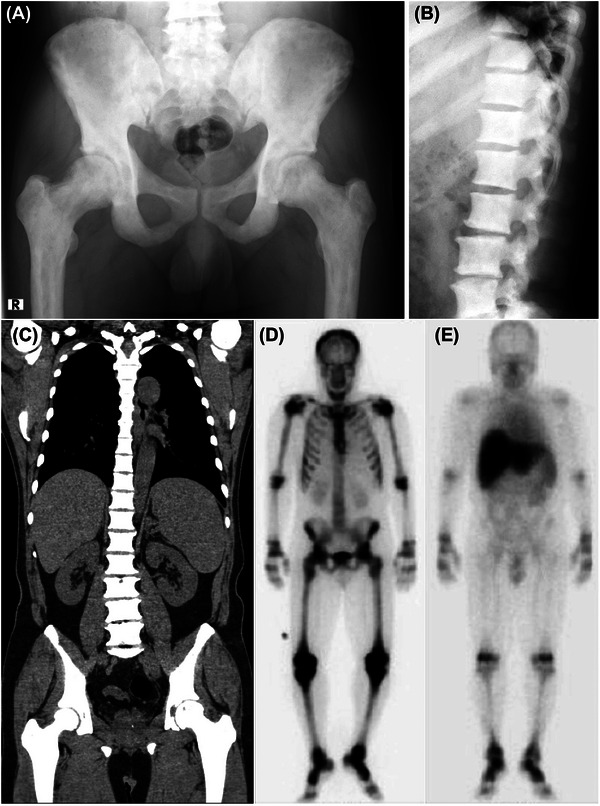
Radiological findings. Plain radiography showed marked calcification of the pelvis (A) and lumbar vertebrae (B). Computed tomography of the body revealed severe systemic osteosclerosis and moderate hepatosplenomegaly (C). Bone scintigraphy showed diffuse high uptakes in systemic bones and joints (D). Bone marrow scintigraphy demonstrated marked accumulation in the liver and spleen, suggesting extramedullary hematopoiesis (E).

Plain radiography showed systemic osteosclerosis, especially in the pelvis, femurs (Figure 1A), and vertebrae (Figure 1B). Computed tomography revealed a marked reduction of the bone marrow space in the humeri, ribs, vertebrae, pelvis, and femurs, in addition to moderate hepatosplenomegaly, but no enlargement of lymph nodes (Figure 1C). Bone scintigraphy with technetium‐99 m hydroxymethylene diphosphonate showed diffuse high uptakes in the spine, pelvis, bilateral shoulder joints, humeri, elbow joints, knee joints, and ankles, which was compatible with systemic osteosclerosis (Figure 1D). Bone marrow scintigraphy with Indium‐111 chloride demonstrated marked accumulation in the liver and spleen but not in the skull, spine, and pelvis, suggesting a marked increase in extramedullary hematopoiesis (Figure 1E).

**FIGURE 2:**
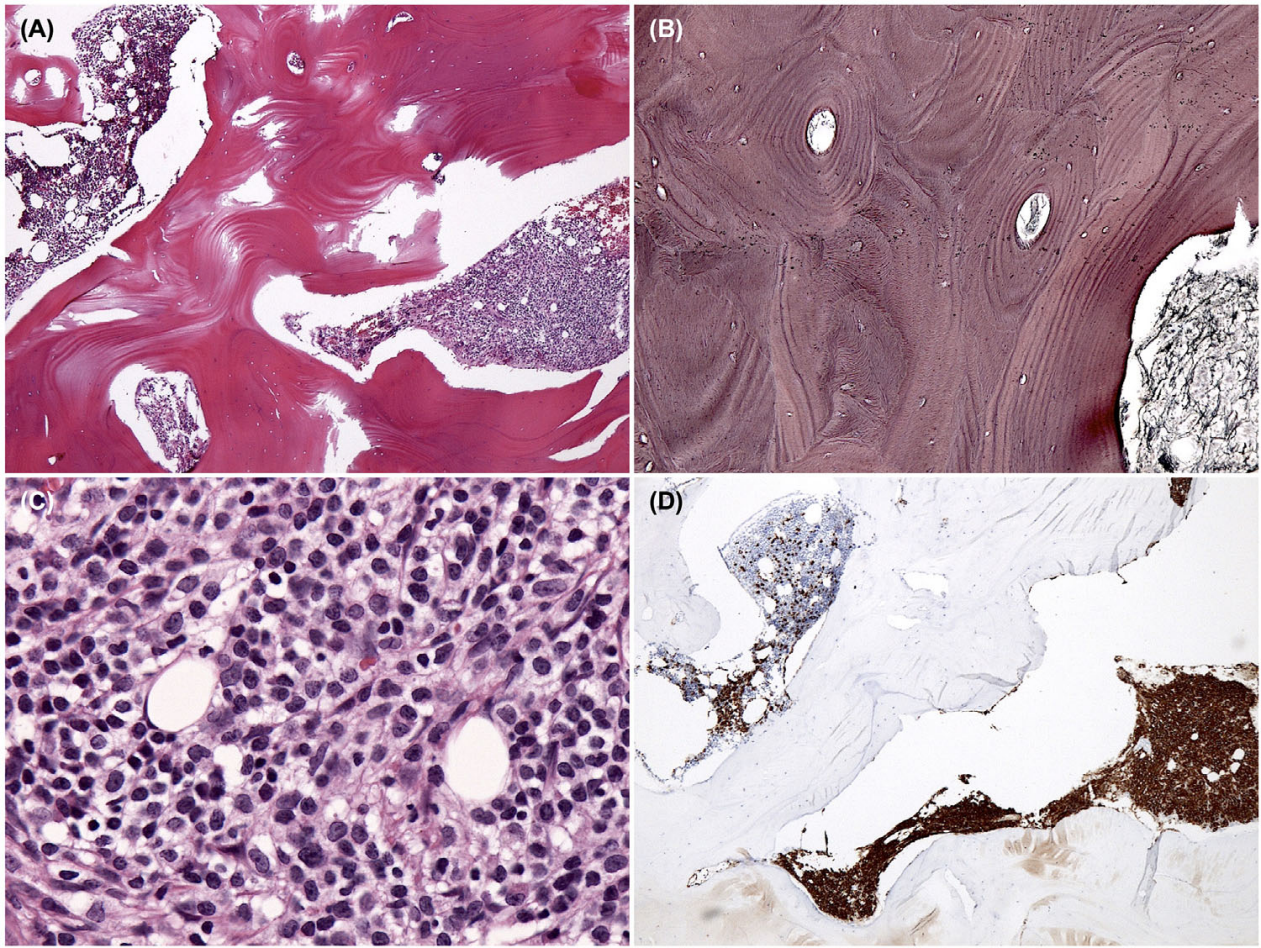
Pathological findings of the bones. Pathological examination of the pelvic bone revealed hypertrophy of the cortical bone (A, original magnification, x40; hematoxylin and eosin [HE] staining), but the lamellar pattern of the bone was regular (B, x400; reticulin staining). There were focal infiltrations of abnormal lymphoid cells in the marrow space (C, x400; HE staining), and these cells were positive for CD20 (D, x40; immunohistochemical staining).

Because the iliac bone itself was extremely hard, a trephine biopsy could not be done and a surgical bone biopsy of the iliac crest was performed. Pathological examination revealed marked hypertrophy of the cortical bone and a decrease of osteoclasts around the bone trabeculae (Figure 2A). The structure of the bone cortex was normal, and there were no abnormal findings of bone remodeling such as disorganized cement lines and mosaic patterns (Figure 2B). Notably, a focal proliferation of atypical lymphocytes was observed in the bone marrow (Figure 2C). These lymphoid cells were positive for CD20 (Figure 2D) and CD79a by immunohistochemistry. There was no evidence of fibrosis of the bone marrow. Based on these findings, we made a diagnosis of systemic osteosclerosis associated with PBML.

To explore the pathophysiological mechanism of osteosclerosis, we examined biochemical markers of bone metabolism. Bone formation markers such as serum bone‐specific ALP and osteocalcin were elevated at 38.6 μg/L (3.7–20.9 μg/L) and 31.8 ng/ml (3.1–12.7 ng/ml), respectively. Urinary markers for bone resorption such as *N*‐terminal crosslinking telopeptide of type I collagen and deoxypyridinoline were markedly increased at 533 nmol BCE/mmol Cr (12.4–71.6 nmol BCE/mmol Cr) and 44.0 nmol/mmol Cr (2.1–5.4 nmol/mmol Cr), respectively. Elevation of both bone formation and resorption markers indicated high turnover of bone metabolism. Serum level of osteoprotegerin, which inhibits osteoclast differentiation and function by interrupting the interaction between receptor activator of NF‐kappa B (RANK) and RANK ligand [13], was elevated at 4.5 pmol/L (0.55‐2.35 pmol/L) as measured by an enzyme‐linked immunosorbent assay (Biomedica, Vienna, Austria).

He was treated with combination chemotherapy consisting of cyclophosphamide, doxorubicin, vincristine, prednisone, and rituximab (R‐CHOP). After six cycles of the treatment, his hip joint pain was relieved and pancytopenia was completely recovered. The serum level of sIL‐2R was reduced to 426 U/ml and the elevated bone metabolic markers were nearly normalized, but no improvement in bone lesions was observed on radiological examination. Positron emission tomography showed no abnormal accumulation and a complete response was obtained. He received autologous stem cell transplantation (ASCT) in the first remission and was followed up without treatment, but relapsed four years later. He received salvage chemotherapy and the second ASCT but relapsed two years later. He is now undergoing rituximab‐based therapy.

## 3 DISCUSSION

PBML tends to have a poor prognosis than other types of lymphoma, and no effective treatment has been established [11]. Only R‐CHOP followed by ASCT has been reported to be effective for a long‐term period [14]. This patient also received the same treatment, but the duration of efficacy was limited. Further treatment strategies are needed to improve the prognosis of PBML.

In regard to the mechanism of osteosclerosis, megakaryocytes and secreted factors as well as osteoprotegerin derived from endothelial cells have been implicated in myeloproliferative diseases [15, 16]. However, the pathogenesis of osteosclerosis has not been clarified in other hematological malignancies. Several case studies have examined the possible role of osteoprotegerin, and these cases are summarized in Table 1. Sato et al. have reported a case of acute megakaryocytic leukemia with osteosclerosis showing that interleukin 11, a factor derived from leukemic cells, induced the expression of osteoprotegerin in osteoblast MG63 cells in vitro [5]. Leung et al. have reported a case of hairy cell leukemia with osteosclerosis with high serum osteoprotegerin levels [10]. The present case of PBML also showed an increase in serum osteoprotegerin levels. Thus, certain types of hematological tumor cells induce the expression of osteoprotegerin in the bone marrow microenvironment, which might lead to the inhibition of bone resorption and enhancement of bone formation.

**TABLE 1:** Reported cases of osteosclerosis associated with hematological malignancies in which the role of osteoprotegerin was examined

**Reference**	**Age**	**Gender**	**Diagnosis**	**Site of osteosclerosis**	**Serum level of OPG** **^a^**
Sato et al. [5]	34	M	AMKL	Vertebrae, pelvis, and tibia	N.D.^b^
Leung et al. [10]	67	M	HCL	Vertebrae, pelvis, and femurs	11.5 pmol/L
The present case	50	M	PBML	Vertebrae, humeri, ribs, pelvis, and femurs	4.5 pmol/L

Abbreviations: AMKL, acute megakaryocytic leukemia; HCL, hairy cell leukemia; M, male; N.D., not described; OPG, osteoprotegerin; PBML, primary bone marrow lymphoma.

^a^ Reference range, 0.55–2.35 pmol/L.

^b^ Addition of the culture medium of the patient's leukemic cells enhanced the expression level of osteoprotegerin mRNA in human osteoblast MG63 cells, and this effect was abrogated by the pretreatment with anti‐interleukin‐11 neutralizing antibody in vitro.

Further investigation of the mechanism of osteosclerosis is needed, and it is important to keep hematological malignancies in mind as a differential diagnosis in case of unexplained osteosclerosis.

## AUTHOR CONTRIBUTIONS

Shuji Ozaki, Tsutomu Iima, Etsuko Sekimoto, Hironobu Shibata, Toshio Shigekiyo, and Yukio Higuchi treated the patient. Takanori Hirose performed pathological examinations. Shuji Ozaki wrote a draft, and proofread the manuscript.

## CONFLICT OF INTEREST

The authors declare no conflict of interest.

## ETHICS STATEMENT

All procedures in this study were performed in accordance with the principles of the Declaration of Helsinki and the institutional guidelines.

## PATIENT CONSENT STATEMENT

Written informed consent was obtained from the patient and his family.

## Data Availability

The data that support the findings of this study are available from the corresponding author upon reasonable request.
